# Electroacupuncture Stimulates Remodeling of Extracellular Matrix by Inhibiting Apoptosis in a Rabbit Model of Disc Degeneration

**DOI:** 10.1155/2015/386012

**Published:** 2015-02-11

**Authors:** Guo-fu Huang, Jing Zou, Jing Shi, Dong-you Zhang, Hong-fen Peng, Qi Zhang, Yu Gao, Bo-yi Wang, Tang-fa Zhang

**Affiliations:** ^1^Department of Neurobiology, School of Basic Medicine, Tongji Medical College of Huazhong University of Science and Technology, 13 Hangkong Road, Wuhan 430030, China; ^2^Department of Acupuncture & Moxibustion, Wuhan Hospital of Integrated Chinese & Western Medicine, Tongji Medical College of Huazhong University of Science & Technology, 215 Zhongshan Road, Wuhan 430022, China

## Abstract

The present study was designed to determine whether EA stimulates remodeling of extracellular matrix by inhibiting apoptosis in degenerated disc. 40 rabbits were randomly assigned to one of the four groups. Animal model was established by a loading device. Magnetic resonance imaging and Pfirrmann's classification were obtained to evaluate both the model and the EA treatment on disc degeneration. The ultrastructure of discs was observed by TEM. Apoptosis involvement was determined with TUNEL staining and western blot for the protein expression of Bax and Bcl-2. The results indicated that EA intervention decreased the MRI grades. TEM analysis showed an apparent remodeling and rearrangement of disc ECM after EA intervention for 28 days. The number of TUNEL-positive cells in the EA group was significantly lower than that in the compression group. The protein expression demonstrated an antiapoptosis effect mediated by EA. Increased expression of Bcl-2 proteins and reduced Bax protein expression were detected after 28 days treatment. It was concluded that antiapoptosis pathway probably participates in the mechanism of EA stimulating the remodeling of ECM in disc degeneration.

## 1. Introduction

Intervertebral disc degeneration (IVDD) is a major cause of many spinal disorders [1, 2]. Degenerated disc can lead to herniations, radiculopathy, myelopathy, spinal stenosis, and degenerative spondylolisthesis, which can cause acute or chronic pain. Low back pain is one of the most frequent conditions requiring medical care and work disability, and 70–85% of all people have back pain at some point in their life [3].

The pathophysiology of degeneration is not completely understood but the consensus is that intervertebral disc degeneration is characterized by ECM decrease. Previous studies were able to demonstrate that the collapse or insufficient rearrangement of a functional ECM equilibrium was strongly associated with apoptosis of IVD cells and its characteristic signaling switch-points [4, 5]. Also, more and more evidences indicating that apoptosis plays an important role in human degenerative diseases. It begins as early as the second decade of life [6] and acted as a quality control mechanism for the maintenance of tissue homeostasis by eliminating defective cells [7].

Acupuncture, which originated in China, was recognized by the World Health Organization as an effective treatment for pain relief and lumbar disc diseases. It was also reported [8] that electroacupuncture (EA) inhibits AF cell apoptosis via the mitochondria-dependent pathway and upregulates Crk and ERK2 expression. In a recent study by our group [9], EA increased ECM content and shows evidence of regenerative potential in degenerated intervertebral discs, as evaluated by protein expression and magnetic resonance imaging. In this study we aimed to detect whether EA stimulates remodeling of ECM by inhibiting apoptosis.

## 2. Materials and Methods

### 2.1. Animals

All animal procedures were performed under the approval and guidance of the Animal Care and Use Committee at Wuhan Hospital of Integrated Chinese & Western Medicine, affiliated to Tongji Medical College of Huazhong University of Science & Technology. A total of 40 New Zealand skeletally mature white rabbits (3.5–4 kg) were used for the study. The rabbits were randomly assigned to four groups; ten for each group was given different interventions at 28-day and 56-day time point [10, 11]. Both the compression group (*n* = 10) and the EA group (*n* = 10) were firstly loaded for 28 days using a custom-made external compression device to stimulate disc degeneration. After 28 days loading time, five in the compression group were killed and the tissue was harvested, the other five using the same device for another 28 days. In EA group, tissue was harvested for five rabbits, and the other five received EA administration for 28 days after removal of the external device. In sham compression group (*n* = 10), the rabbits received surgical preferment, but the lumbar body was only punctured without previous loading for 28 days (*n* = 5) or 56 days (*n* = 5). Ten rabbits, which served as controls, were normally fed without surgical preferment for 28 days (*n* = 5) or 56 days (*n* = 5).

### 2.2. Surgical Procedure

**Figure 1:**
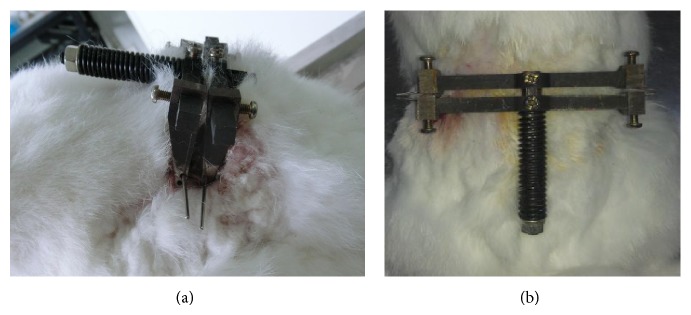
Degenerated model induced with a load. Different perspectives ((a) and (b)) of external dynamic compression device attached to the rabbit lumbar spine (see [9]).

Rabbits were anesthetized with 10% chloral hydrate administered via the marginal ear vein. Through a dorsal approach to the lumbar spine, the custom-made external device was attached to two K-wires (1.5 mm diameter) inserted into the vertebra body L4 and L5 parallel to the adjacent study disc by use of a variable-speed electric drill [12]. After the wound was closed, axial compression to the disc was created by a spring within the device to produce a disc compression force of 200 N to induce disc degeneration (Figure 1). The sham compression group was performed the same way, but the external compression device was placed in situ without application of compressive force.

### 2.3. Magnetic Resonance Imaging

Magnetic resonance imaging (MRIs) was obtained for each group at days 28 and 56. Imaging was taken at 30 minutes after removal of the external fixateur to establish new hydration equilibrium of the disc. MRIs were performed with a 3.0 T imager (GE, American) with a synergy spine coil receiver. T2-weighted sections in the sagittal plane were obtained in the following settings: fast spin echo sequence and time to repetition (TR) 2200 milliseconds; time to echo (TE) 70.7 milliseconds; matrix 336 (h) ∗ 512 (v); field of view 120 mm; 8 excitations; section thickness 2 mm; gap 0.2 mm (T1: TR 375; TE 15; matrix 304 (h) ∗ 512 (v); 18 excitations). The Pfirrmann's classification [13] was used for disc degeneration grading from grade 1 to 5 (1 = normal, 2 = mild degeneration, 3 = moderate degeneration, 4 = severe degeneration, and 5 = advanced degeneration).

### 2.4. EA Treatment

The rabbits in the EA treatment group received EA administration on the Ex-B2 (paravertebral point of L4 and L5 level on both sides) once every day, starting at the second day after the device was removed and lasted for 28 days. Four acupuncture needles were inserted into 4 acupoints that correspond to Ex-B2 in the rabbits; EA (1 mA and 0.4 or 0.6 ms) was administered at 2 or 15 Hz for 30 minutes. Current was delivered with a modified current-constant Han's Acupoint Nerve Stimulator (Bei-jing, China). Ex-B2 was chosen according to the traditional Chinese medicine meridian theory and the effective use in reducing pain. During EA treatment, each rabbit was placed under an inverted clear wooden box (approximately 40 × 25 × 40 cm) but was neither restrained nor given any anesthetic. The animals remained awake and still during EA treatment and showed no evident signs of distress.

### 2.5. Tissue Preparation

After different intervention, the lumbar disc was harvested for examination of each group, including complete annulus fibrosus and nucleus pulposus. Using a vertical midline incision, the disc was divided into 2 symmetric parts. One part was immediately quick-frozen in liquid nitrogen for protein expression; the second part was used for morphology study and apoptosis analyze.

### 2.6. Transmission Electron Microscopy

Samples of NP and AF were fixed in a mixture of 2% glutaraldehyde with phosphate buffer (pH 7.4) for two hours, subsequently postfixed in a 1% solution of osmium tetroxide with 1.5% potassium ferrocyanide. Following being dehydrated in graded alcohols, the samples were embedded in Epon. Ultrathin sections were prepared and contrasted with uranyl acetate and lead citrate. Sections were examined using the electron microscopy by FEI Tecnai company with an accelerating voltage of 160 kV.

### 2.7. TUNEL Assay

The tissue specimens were fixed in 10% neutral formalin for 24 h and subsequently dehydrated, cleared, and embedded in paraffin using standard procedures. Deparaffinised and rehydrated sections were immersed in 3 mL/L H_2_O_2_ for 30 min at room temperature and digested with proteinase K for 15 min at 37°C. The sections were immersed in 1 g/L Triton-100 and then incubated with TUNEL mixture for 1 hour at room temperature and then stained with 0.4 g/L DAB and treated with 3 mL/L H_2_O_2_ for 10 min and with hematoxylin for 1 min. Total and TUNEL-positive disc cells were counted below five noncontinuous high-power fields (magnification, ×400). The percentage of TUNEL-positive disc cells compared with total disc cells was then calculated.

### 2.8. Western Blotting Analysis

Total protein was extracted from the tissue in RIPA lysis buffer (containing protease and phosphatase inhibitor mixtures) by using a tissue homogenizer, followed by clearing tissue debris by centrifugation at 13000 rpm at 4°C for 20 min. Fifty micrograms of protein was loaded per lane and separated by 10% SDS-PAGE gel electrophoresis and then transferred onto PVDF membranes. Blocking was carried out in 5% nonfat dry milk in Tris-buffered saline (TBS) containing 0.1% Tween-20 for 1 h at room temperature. The membranes were incubated with primary antibody rabbit anti-Bax (diluted 1 : 300, Bioss, Beijing, China); anti-Bcl-2 (diluted 1 : 300, Bioss, Beijing, China), overnight at 4°C and with secondary antibody (1 : 40000 dilution of goat anti-rabbit) conjugated to horseradish peroxidase (Boster, Wuhan, China) for 1 h at room temperature on the following day. Immunoblotting signal was detected by ECL (enhanced chemiluminescence) on chemiluminescent films following exposure to an X-ray. For densitometric analyses, the blots were scanned and quantified using BandScan software (Glyko Biomedical, Canada), and the result was expressed as the ratio of target gene immunoreactivity to GAPDH immunoreactivity.

### 2.9. Statistical Analysis

The data collected in the present study were expressed as mean ± standard deviation (Mean ± SD) and analyzed by one-way repeated measures ANOVA to determine difference between two groups. Statistical analyses were performed using the SPSS 11.5 statistical software program (SPSS Inc., Chicago, USA). *P* < 0.05 was considered statistically significant.

## 3. Results

### 3.1. The Effect of EA on MRI Grade Scores in Disc Degeneration

We firstly evaluated whether the loading device induce the degeneration model successfully. The MRI assessment showed that the signal intensity of the nucleus pulposus decreased progressively during the 28-day compression period; the healthy and compressed discs are clearly different on T2-weighted image. The result was consistent with previous research [14]. No significant difference was seen between the control and sham groups at any point. After EA intervention, a slight recovery of signal intensity was observed.

**Figure 2:**
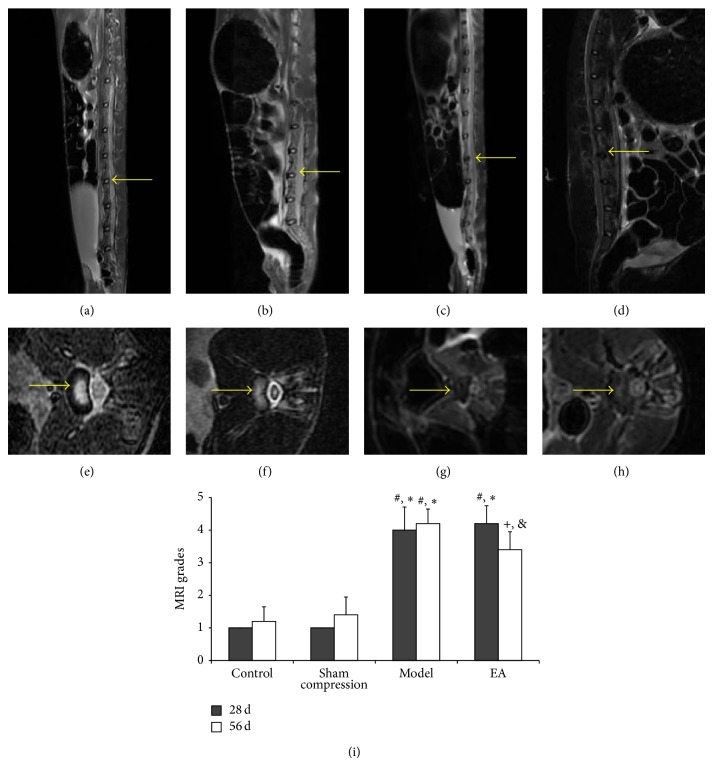
MRI image of the lumbar spine and the Pfirrmann's classification grade. Representative T2-weighted sagittal MRI of the nucleus pulposus at the 56-day time point shows different signal intensity in control group (a), sham group (b), compression group (c), and EA group (d). (e), (f), (g), and (h) are the corresponding MRI axial scan, respectively, to (a), (b), (c), and (d). Different signal intensity in the disc is depicted with arrows. Change in disc degeneration of four groups (i). Pfirrmann's classification based on disc height and signal intensity from grade 1 to 5 was used to grade the disc degeneration of the rabbit discs. Data are expressed as the mean ± SD (ANOVA); ^*^ *P* < 0.05, compared with the control group; ^#^ *P* < 0.05, compared with sham compressed group; ^+^ *P* < 0.05, compared with compression group in 56 days and the control group; ^&^ *P* = 0.046, compared with 28 days in EA group (see [9]).

According to the Pfirrmann's MRI grade scores, which indicate the degree of disc degeneration, grade IV degenerative changes were firstly detected at 28-day postcompression; grade IV or V was detected 56 days after loading. The control and sham groups remained relatively constant during the 28- or 56-day period, with grade I on T2-weighted imaging. Compared to the compression group and EA group in 28 days, the degree of degenerated disc at 56-day time point in EA group was characterized by grade III or IV (*P* < 0.05) (Figure 2).

### 3.2. The Effect of EA on the Remodeling of ECM in Disc Degeneration

**Figure 3:**
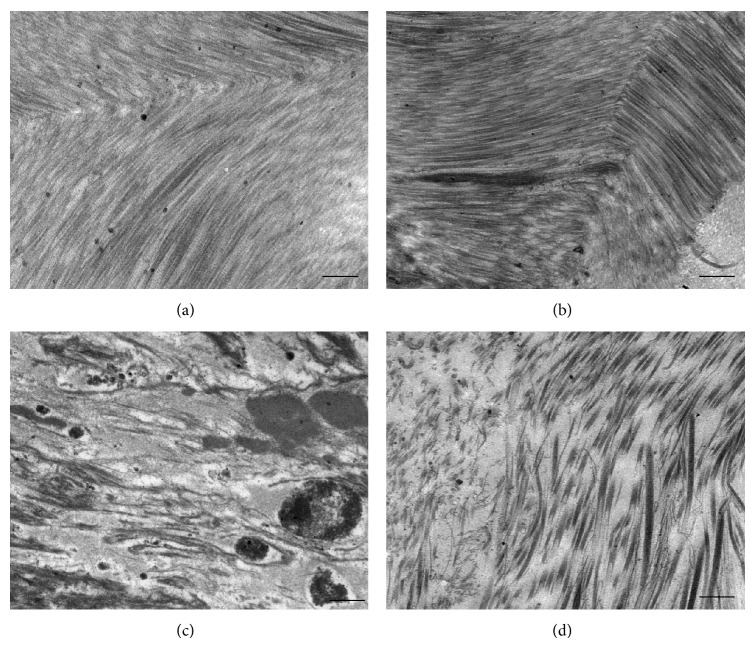
TEM observation of ECM. Representative electron photomicrographs showed the ultrastructural change of ECM in rabbit disc. The collagenous fibrils of control (a) and the sham compressed annulus fibrosus (b). The collagenous fibrils of degenerative rabbits in compression group (c). The remodeling and rearrangement of ECM in EA group (d). Scale bars: 2 *μ*m.

It was noted previously [15] that human disc consisted of sparse AF or NP cells and a large amount of dense ECM, such as the collagenous fibrils and glycosaminoglycans. By ultrastructure examination (Figure 3), the changes of annulus fibrosus were significantly different after loading or EA treatment. Transmission electron microscopy showed that the ECM was compact lamellar structure with a certain order. The collagenous fibrils of normal and sham compressed AF were tightly and orderly arranged at 28- or 56-day time point. While in the compression group, we found that the severely degenerative AF was cracked and randomly arranged after 56 days compression. For the rabbits in EA group, the collagenous fibrils were in a loose order but obviously rearranged compared with the compression group. Morphological studies indicate that EA induced the remodeling of ECM in degenerated disc.

### 3.3. The Effect of EA on Apoptosis Assessed by TUNEL Staining in Disc Degeneration

**Figure 4:**
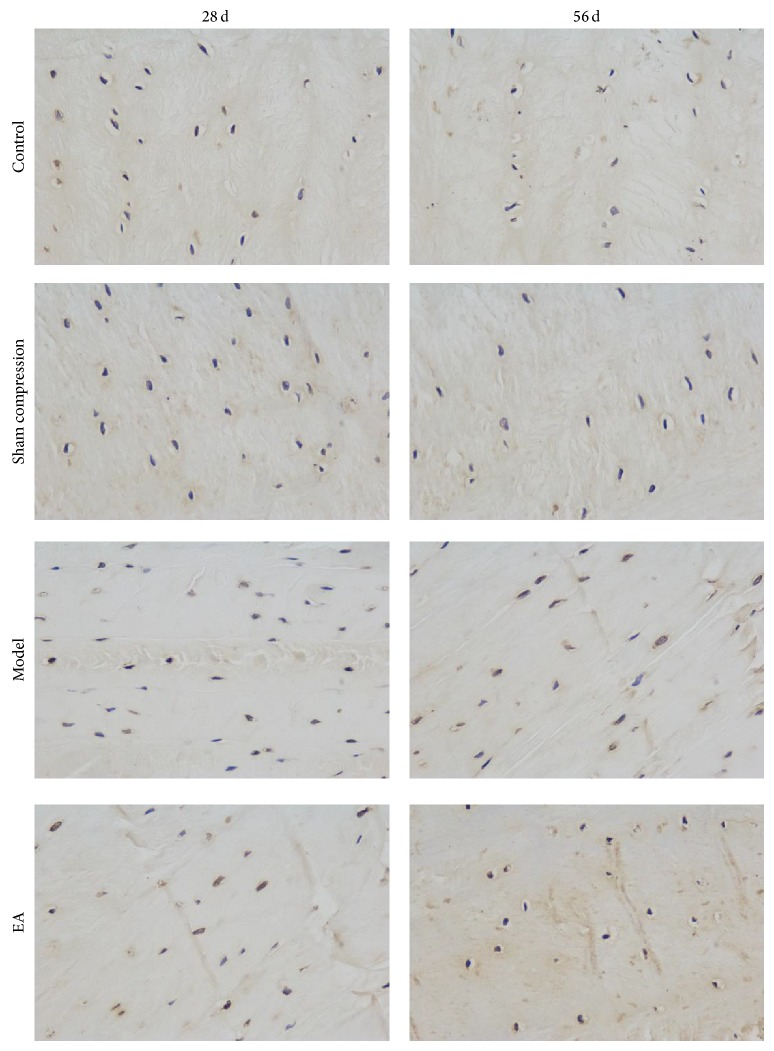
Representative disc cell apoptosis in four groups. The percentage of TUNEL-positive disc cells was calculated.

TUNEL staining allows the detection and quantification of apoptosis at a cellular level based on the labeling of free 3-OH terminals created during double-strand and single-strand cleavage of genomic DNA. In control and sham compression group specimens, very few apoptotic cells were observed under the microscope. In compressed discs, there was a significant increase in apoptosis in the nucleus pulposus and annulus. There was obvious disruption of the nuclear membrane and apoptotic bodies were seen in the disc loaded for 56 days. However, the number of TUNEL-positive cells in the EA group was significantly lower than that in the compression group (Table 1, Figure 4).

### 3.4. The Effect of EA on Apoptosis Regulatory Protein in Disc Degeneration

**Figure 5:**
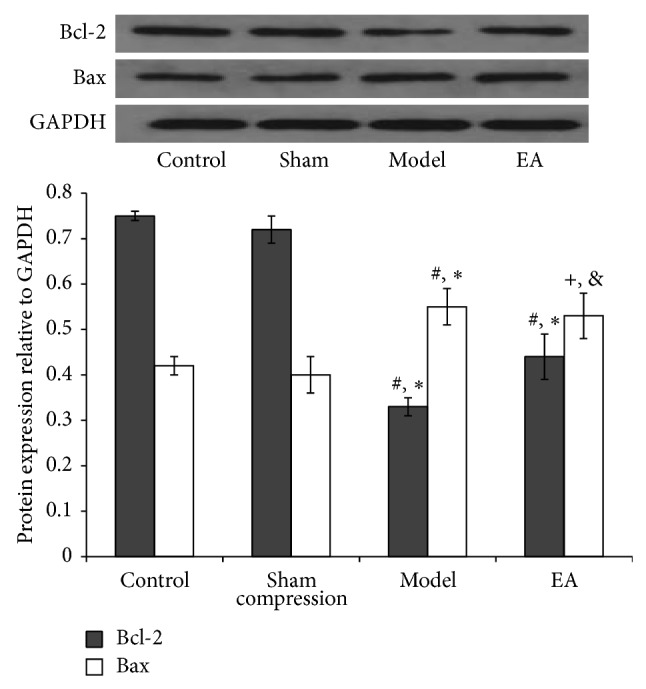
Representative western blot analyses showing the Bcl-2 and Bax protein levels in 28 d of four groups, 5 samples for each group. GAPDH was analyzed as housekeeping gene. Western blotting was performed 5 times to evaluate the protein expression of four groups. ^*^ *P* < 0.05, compared with the normal control; ^#^ *P* < 0.05, compared with sham group; ^+^ *P* > 0.05, compared with the compression group; ^&^ *P* > 0.05, compared with the compression group.

**Figure 6:**
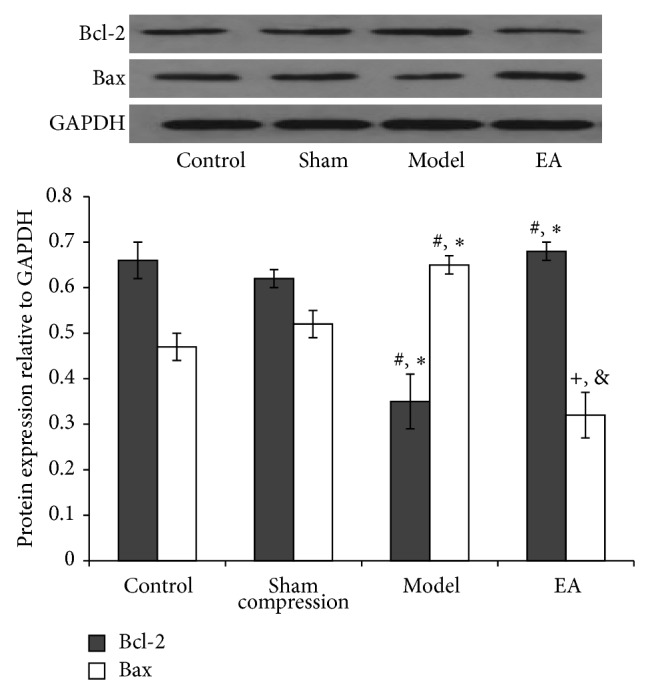
Representative western blot analyses showing the Bcl-2 and Bax protein levels in 56 d of four groups, 5 samples for each group. GAPDH was analyzed as housekeeping gene. Western blotting was performed 5 times to evaluate the protein expression of four groups. ^*^ *P* < 0.05, compared with the normal control; ^#^ *P* < 0.05, compared with sham group; ^+^ *P* < 0.05, compared with the compression group; ^&^ *P* < 0.05, compared with the compression group.

Bcl-2 family proteins are key regulators of mitochondria-mediated apoptosis and include antiapoptotic members such as Bcl-2 and proapoptotic members such as Bax. In the present study, western blot analysis demonstrated that no significant changes were found between control and sham compression groups at any time point. Compared with the two groups, the relative expression levels of Bcl-2 protein in 28 (Figure 5) and 56 (Figure 6) days were apparently decreased in the compression group (*P* < 0.05). Following EA intervention, the expression was considerably higher than the compression group in the 56 days (*P* < 0.05). A trend to stimulate an antiapoptosis effect was found in EA group. The result of protein Bax was in contrast to that observed in Bcl-2. High expression levels of Bax protein in 28 (Figure 5) and 56 (Figure 6) days were detected in the compression group. After EA treatment for 28 days, the expression decreased considerably than the compression group in the 56 days (*P* < 0.05).

## 4. Discussion

Many clinicians and researchers believe that disc degeneration is the predominant source of low back pain, as the degenerative changes can lead to nerve root compression, resulting in radiculopathy [16]. Over the past decade, the mechanisms of IVD degeneration have been partly documented in terms of biomechanics [17] and cell apoptosis [18, 19]. Recent studies have shown that apoptosis plays a key role in ECM decrease and a high incidence of apoptotic cells is observed in human aged and degenerated discs [20]. Therefore, treatment targeting programmed cell death interception will be a potential direction for preventing degeneration process. In a recent research by our group, we found that acupuncture is an effective approach among the alternatives. Although acupuncture has been practiced for over 4,000 years, it has been difficult to establish its biological basis. This study provides new information about the mechanisms underlying the antiapoptosis effect of EA on degenerated ECM remodeling.

To study disc degeneration or disc regeneration, establishment of an in vivo animal model is essential. Mechanical stress is one of the key contributors to intervertebral disc degeneration [21]. In the rodent tail model by Lotz and colleagues [22], static compression initiates apoptotic cell death in the inner AF, cartilage endplates, and then NP. Previously we reported a rabbit model of disc degeneration induced by a self-made mechanical loading device, which mimics extracellular matrix metabolic imbalances in disc degeneration [9]. The observed biological mechanisms are consistent with human characteristic [23, 24]. This similarity conveys the primary advantage for longitudinal investigation.

In this study, the degeneration model and the effect of EA were evaluated by MRI method. In contrast to sham or controlled discs, pictures of rabbit lumbar spines in compression group showed a significant decrease of nucleus pulposus hydration after 28 days of compression thus indicating that the static compression model may be associated with cell death or nutrient deprivation. During aging and degeneration, surviving cells are not synthetically inactive but are, rather, producing inappropriate matrix products [25]. For the rabbits in EA group, EA intervention resulted in a number of slowly progressive and reproducible MRI changes over 28 days. On the other hand, the Pfirrmann's classification system was adapted to assess the effect of EA. We found that the degeneration grade on MRI was slight but significantly decreased after EA treatment.

The disc is a specialized biomechanical structure, including the annulus fibrosus (AF) made of tough concentric lamellae rich in collagen and the nucleus pulposus (NP) made of proteoglycans (PG), water, and collagens. Intervertebral discs are avascular pads of fibrocartilage with a low cell density and a limited ability to adapt to mechanical demands. In normal discs, the well-organized concentric collagen lamellae in AF are best appreciated under polarized microscopy; in degenerative discs, the collagen fibers in AF are disorganized. Biochemically, disc degeneration is known by decreased expression of ECM, such as aggrecan [26] shifts in the collagen expression [27] and changes in collagen cross-linking indicative of increased matrix turnover [28]. Increasing attention has been paid to the restoration and remodeling of the ECM integrity by cell therapy, gene engineering, and other methods [29, 30]. Our results present direct morphologic evidence that EA intervention stimulated remodeling of ECM. The apparent tissue remodeling in degenerated discs coincides with significant cell matrix changes. It was characterized by the decrease of collagen content, alteration of collagen distribution, and an increase of collagen cross-links and a decrease of proteoglycan levels. The present TEM observation showed that the annulus matrix in normal and sham disc was tightly and orderly arranged. While in the severely degenerative AF, ECM were cracked and randomly arranged. EA was able to halt or even reverse the collapse or insufficient rearrangement of ECM.

Apoptosis in IVD degeneration was initially reported by Gruber in 1998. More particularly, studies investigating the regulation of cartilage matrix suggested an important coordinating role of Bcl-2 in the regulation of collagen type II [31]. In order to study whether EA triggered remodeling of annular ECM by inhibiting apoptosis, we made a further study about the expression of Bcl-2 protein kindred in disc degeneration. It has been demonstrated that apoptosis is a procedure of death adjusted by a cluster of apoptotic genes and protein, in which the bcl-2 gene kindred is an important apoptosis adjusting gene. Bcl-2 protein is located in mitochondrial membrane, endoplasmic reticulum, and nuclear membrane. As it could prolong the life of cells, it has been generally accepted as an antiapoptosis gene [32–34]. Bax is a member of bcl-2 gene family; it could form a dimer with bcl-2 to inhibit its function [35–37]. The ratio of Bax to Bcl-2 is therefore critical in determining the fate of cells. A previous comparative study concluded that increased proapoptotic proteins are an indication of static compression induced ECM decrease and degeneration [38]. Overexpression of antiapoptotic proteins through the mitochondrial pathway (e.g., Bcl-2) may represent a specific, effective molecular treatment option in degenerative disc disease. The results described in the present research are in agreement with several earlier studies. We found that EA treatment increased the expression of Bcl-2 proteins and reduced the Bax protein expression. This finding indicates that EA inhibits apoptosis by affecting the Bax/Bcl-2 ratio.

In summary, our study investigated the antiapoptosis effect of EA treatment on a degenerated disc rabbit model. We confirmed that EA significantly reduced Pfirrmann's MRI grade scores and improved ultrastructure change in degenerated disc. The underlying mechanism of EA delaying degeneration may be due to the regulation of EA in the balance of Bcl-2 and Bax. Our results provide a basis for further studies to determine the specific signaling pathways of EA delaying IVD degeneration.

## Figures and Tables

**Table 1:** TUNEL-positive rate in four groups (%).

Group	28 d	56 d
Control	16.27 ± 2.18	15.51 ± 3.06
Sham	17.64 ± 1.98	18.86 ± 2.47
Compression	69.95 ± 5.04^∗,#^	74.37 ± 4.73^∗,#^
EA	65.42 ± 3.97^∗,#^	43.36 ± 7.25^+,&^

Data are expressed as the mean ± SD (ANOVA); ^*^ *P* < 0.05, compared with the control group; ^#^ *P* < 0.05, compared with sham compressed group; ^+^ *P* < 0.05, compared with compression group in 56 days and the control group, ^&^ *P* < 0.05, compared with the 28 days in EA group.
